# Late Penile Urethral Involvement of Urothelial Carcinoma After Radical Cystectomy: Diagnostic Challenge in Distinguishing Urethral Recurrence From Metastatic Disease

**DOI:** 10.1155/cris/4918822

**Published:** 2026-10-09

**Authors:** Vanessa Talavera Cobo, Mario Daniel Tapia Tapia, Andrés Calva López, Carmina Alejandra Muñoz Bastidas, Luis Labairu Huerta, Daniel Sánchez Zalabardo, José Enrique Robles García, Bernardino Miñana López

**Affiliations:** ^1^ Urology Department Clínica Universidad de Navarra Avenue Pío XII, 36 Pamplona 31008 Navarra Spain cun.es; ^2^ Urology Department Clínica Universidad de Navarra St. Marquesado de Santa Marta 1 Madrid 28027 Spain cun.es

**Keywords:** late recurrence, metastasis, penectomy, perineal mass, tunica albuginea, urethral recurrence, urothelial cancer

## Abstract

Penile involvement of urothelial carcinoma is a rare clinical entity typically arising from primary pelvic tumours and is usually associated with advanced disease and poor prognosis. However, distinguishing between true penile metastasis and late urethral recurrence is challenging, particularly in patients with urethral preservation after radical cystectomy (RC). We report the case of a 77‐year‐old male with a history of high‐grade invasive urothelial carcinoma (pT2a N0) with concomitant carcinoma in situ (CIS), treated with RC and uretero‐ileal urinary diversion with urethral preservation. Ten years after surgery, the patient presented with a painful mass at the base of the penis accompanied by solid urethral discharge. Biopsy of the urethral content confirmed high‐grade papillary urothelial carcinoma (G3) with invasion of the lamina propria. Multimodal imaging studies demonstrated a solid tumour involving the proximal third of the penile urethra with extension into the corpus spongiosum and tunica albuginea, alongside a suspicious left inguinal lymph node. The patient underwent radical penectomy and left laparoscopic inguinal lymphadenectomy. Histopathological examination confirmed high‐grade invasive urothelial carcinoma of the penile urethra, invading the corpus spongiosum and tunica albuginea (pT2 N0), with free surgical margins. Immunohistochemical profiling was not performed due to limited archival material, preventing definitive distinction between urethral recurrence and metastatic disease. No adjuvant systemic therapy was administered. At 2 years of follow‐up, the patient remains disease‐free on surveillance imaging. This case highlights the unusual late presentation of urothelial carcinoma involving the penis and underscores the diagnostic challenge in distinguishing urethral recurrence from true metastatic disease in the absence of molecular data. Long‐term surveillance, including thorough physical examination, is essential even many years after initial treatment.

## 1. Introduction

Penile metastases are a very rare occurrence, even though the penis is highly vascularised and anatomically adjacent to pelvic organs such as the bladder, prostate and rectum [1]. The primary origins of penile metastases are predominantly genitourinary tumours: 35% from bladder cancer, 30% from prostate cancer, 13% from colon cancer and less than 5% from kidney or testicular cancer [2]. The presence of penile metastases indicates widespread disease and therefore confers a poor prognosis [3].

Nevertheless, not all penile involvement of urothelial carcinoma after radical cystectomy (RC) is indicative of lymphatic or haematogenous metastases. The retained urethra in patients undergoing RC with urethral preservation is still susceptible to recurrence, especially if the original specimen contained carcinoma in situ (CIS) [4, 5]. About 4%–10% of male patients experience urethral recurrence following RC, which can appear years after the initial procedure [4, 5]. Differentiating between distant metastatic spread and urethral recurrence, which indicates locoregional disease potentially treatable with curative intent, has essentially different prognostic and therapeutic implications [4, 5]. However, in clinical practice, this distinction is often difficult and might not be possible without molecular or immunohistochemical analysis.

When penile involvement develops after an abnormally long period of time without disease, the diagnostic challenge is exacerbated. While most penile metastases from bladder cancer appear within the first 18 months of diagnosis or concurrently with the primary tumour [6, 7], late presentations that happen 10 years or more after RC are extremely uncommon and only reported in a few isolated case reports [3, 7]. The standard oncological surveillance paradigm, which usually increases follow‐up in the early post‐operative years and gradually decreases it afterward, is challenged by such cases [5].

This case report presents a unique presentation of penile urethral involvement by high‐grade urothelial carcinoma occurring after 10 years following RC and urethral preservation for muscle‐invasive bladder cancer with associated CIS in a 77‐year‐old man. This report aims to describe the clinical, radiological and pathological features of this case, to assess the challenging diagnosis of differentiating metastatic disease from late urethral recurrence and to contribute to the limited evidence regarding surveillance and treatment decisions in this specific clinical scenario. The lack of clonality studies in this case underscores the significance of prospective tissue banking protocols and draws attention to a common limitation of the current literature.

## 2. Case Presentation

A 77‐year‐old male underwent regular urological follow‐ups due to a past medical history of papillary urothelial carcinoma of the bladder (pT1 G2), initially treated with transurethral resection of the bladder (TURBT) and intravesical instillations of mitomycin C. Subsequent restaging TURBT demonstrated progression to muscle‐invasive urothelial carcinoma. Pelvic magnetic resonance imaging confirmed infiltration of the left bladder wall, extending along the left ureter and perivesical fat invasion.

Given these findings, the patient underwent RC with uretero‐ileal urinary diversion. At the time of RC, the urethra was preserved due to the absence of tumour involvement near the trigone. Intraoperative urethral margins were not submitted separately; only the final pathological examination reported negative urethral margins. Pathological examination revealed high‐grade invasive urothelial carcinoma (pT2a N0) with concomitant CIS and negative urethral margins. No neoadjuvant or adjuvant systemic therapy was administered. The patient was enrolled in a structured post‐operative surveillance programme and remained disease‐free throughout follow‐up.

Ten years after primary treatment, he consulted again, complaining of a painful mass at the base of the penis on the left side, associated with solid urethral discharge (Figure 1). Pathological analysis of the manual expression of urethral solid content revealed high‐grade papillary urothelial carcinoma (G3) with invasion of the lamina propria.

**Figure 1:**
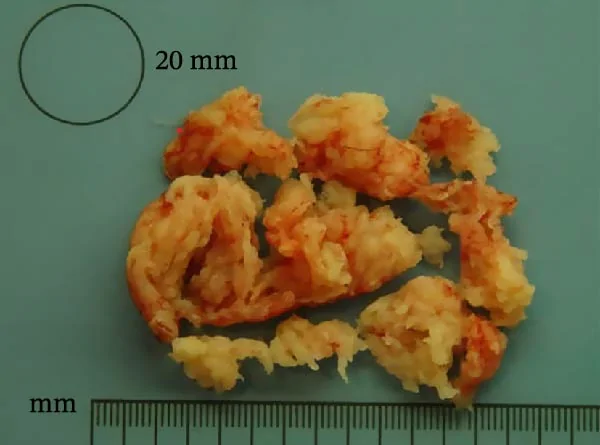
Solid material extracted from the urethra during the physical examination in consultation, submitted to pathology for analysis.

Further imaging studies were performed to determine the extent of the lesion, including penile ultrasound (Figure 2), abdominal computed tomography (Figure 3) and PET‐CT/FDG (Figure 4).

**Figure 2:**
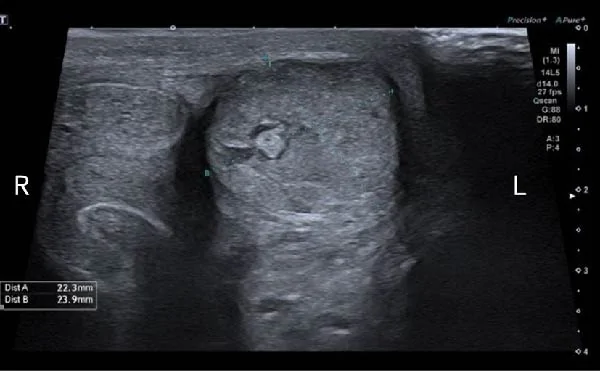
Penile ultrasound depicted a solid tumour in the left *corpus cavernosum*, measuring 33 mm × 24 mm × 24 mm, apparently confined to the *corpus cavernosum* and situated in its base. The tumour exhibited moderate vascularisation.

**Figure 3:**
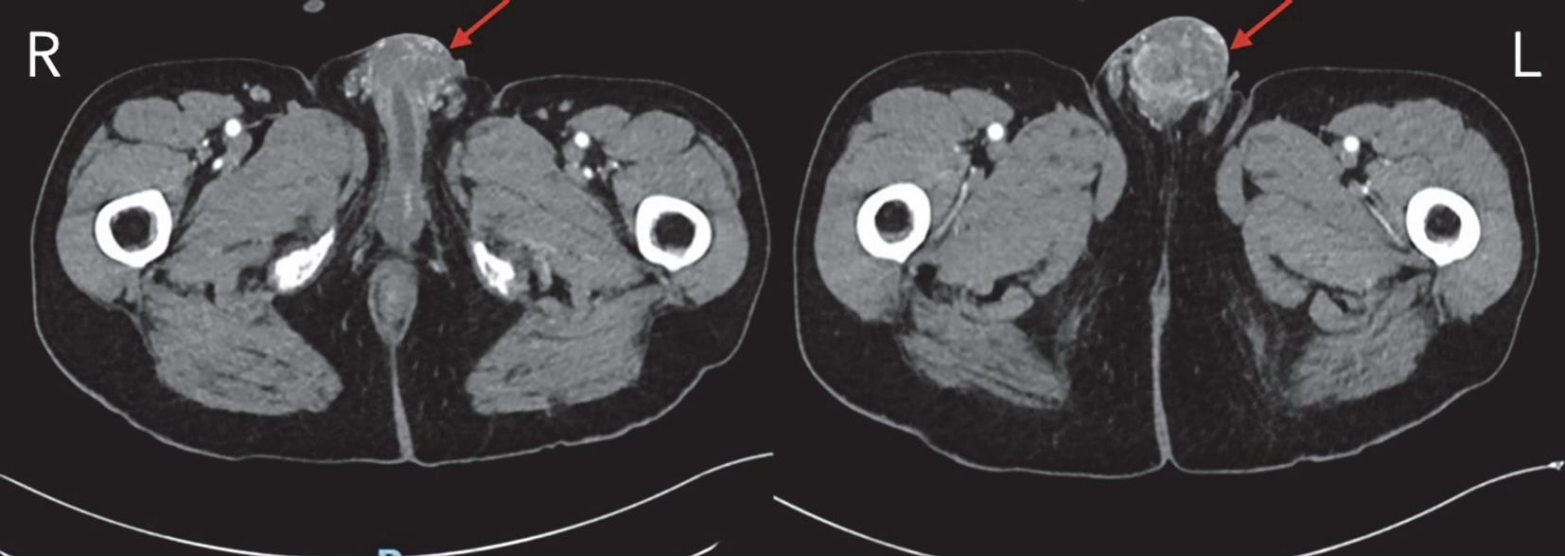
Abdominal computed tomography identified a solid lesion in the left lateral region at the base of the penis measuring 3.6 cm × 3 cm × 5.1 cm, with signs of urethral infiltration, accompanied by proximal urethral dilation up to 8 mm in length.

**Figure 4:**
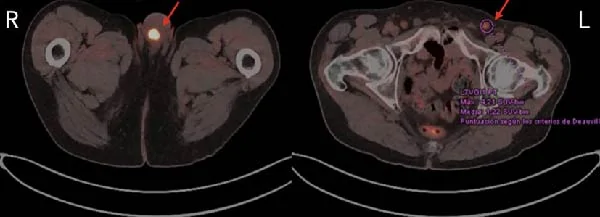
PET/CT‐FDG imaging revealed a hyperactive lesion located on the left base of the penis, consistent with tumour infiltration, associating suspicious left inguinal lymph nodes.

Following interdisciplinary discussion, surgery was chosen as the primary treatment because the lesion appeared localised on imaging, the patient had excellent performance status and no distant metastases were identified. Systemic therapy was deferred pending pathological staging. The patient underwent radical penectomy and left laparoscopic inguinal lymphadenectomy. The decision to perform lymph node dissection was based on the presence of a suspicious left inguinal lymph node on PET/CT imaging, together with the confirmed left‐sided location of the primary lesion on both imaging studies and physical examination. Histopathological analysis confirmed high‐grade invasive urothelial carcinoma of the penile urethra with invasion of the corpus spongiosum and tunica albuginea without lymph node involvement (pT2, N0 AJCC 9th edition urethral staging) (Figure 5). The morphological features were fully consistent with a urothelial origin. Clonality studies and immunohistochemical profiling were not performed at the time of diagnosis, and retrospective analysis was not feasible due to the limited remaining archival material. This represents a major limitation in definitively classifying the lesion as either urethral recurrence or true penile metastasis.

**Figure 5:**
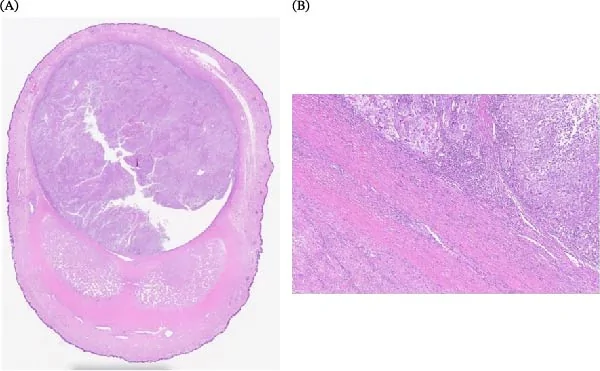
Representative histopathological image showing high‐grade invasive urothelial carcinoma of the penile urethra (pT2, N0), with invasion of the corpus spongiosum and tunica albuginea, and free surgical margins. Haematoxylin and eosin (H&E) stain. (A) Low‐power view (×0.25). (B) Higher‐power view (×8).

No adjuvant systemic therapy was administered following surgery. At the most recent follow‐up, 2 years after surgery, the patient remains disease‐free on surveillance imaging.

## 3. Discussion

Penile involvement is rare, despite the rich vascularisation of the penis and its proximity to pelvic organs like the bladder, prostate and rectum. Research by Youssef et al. [6] indicates that primary tumours typically stem from the bladder and prostate. Furthermore, a comprehensive review by Meairini et al. [7] highlights that only 500 cases of penile involvement had been documented in the literature up until 2012.

The mechanism underlying the dissemination of urothelial carcinoma to the corpora cavernosa remains elusive. Some authors proposed theories including direct tumour spread, venous, arterial or lymphatic dissemination and iatrogenic routes [6]. Among these, retrograde venous dissemination has garnered attention as the primary mechanism [5].

The detection of penile involvement determines a poor prognosis, confirming an advanced stage. The timeframe between the primary tumour diagnosis and the emergence of penile metastases varies widely, spanning from weeks to years [7]. One‐third of penile metastases manifest concurrently with the primary tumour, whereas the remaining two‐thirds emerge within the initial 18 months postdiagnosis [6, 8]. The peculiarity of our case lies in the very late presentation of urothelial carcinoma involving the penis, which occurred a decade after the cystectomy, highlighting that during this period, the patient remained asymptomatic and had no evidence of disease on imaging studies. Although penile metastasis is a recognised entity, the clinical and pathological features in this case raise the possibility of a late urethral recurrence from the preserved urethra. This is supported by the presence of concomitant CIS at the time of cystectomy and the anatomical location of the lesion. Therefore, distinguishing between metastatic disease and urethral recurrence remains challenging in this context. This case also raises the question of whether prophylactic urethrectomy should be considered in selected high‐risk patients, particularly those with concomitant CIS. Although urethral preservation is generally accepted when margins are negative, careful long‐term surveillance is essential.

More than half of penile metastase cases manifest as a 1–2 cm nodule (52%), primarily located at the base of the penis and less commonly in the corpus spongiosum or glans [1]. Priapism occurs in up to 21% of cases, typically due to involvement of the corpora cavernosa, either through compression of venous drainage or thrombosis of cavernous arteries caused by tumour invasion [5]. Controversy surrounds whether malignant priapism indicates a worse prognosis as there is no consensus on this aspect among reviewed articles [2]. In certain instances, perineal pain, cutaneous lesions or urinary obstruction due to urethral compression may be the sole manifestations of penile metastases [2].

Histopathological diagnosis through biopsy is essential for confirming penile involvement and imaging studies play a crucial role in staging the condition; however, they are not conclusive [5].

Patients with a history of urothelial carcinoma should undergo strict and comprehensive follow‐up, including detailed physical examinations, especially of the genital region. While the poor prognosis associated with penile metastases is recognised, timely identification and intervention could potentially change patient outcomes.

The absence of concrete evidence in this domain renders treatment decisions heavily reliant on the surgeon’s expertise and the patient’s overall health. Treatment modalities vary widely, spanning from surgery to systemic therapies, or in some cases, exclusively palliative care [5, 6]. The patient’s general health status and life expectancy serve as critical factors in decision‐making.

According to isolated cases documented in the literature, radical penectomy appears to enhance patient survival, particularly in those with localised lesions [3]. A systematic review conducted by Cocci et al. [9] on penile metastases, encompassing 63 articles, indicates an average survival of 14.5 months, with outcomes being more favourable in cases where primary urothelial tumours were free of malignant priapism. Radical penectomy can be considered in patients experiencing unmanageable perineal pain as a palliative measure.

In the aforementioned case, both treatment strategies converge radical penectomy as a palliative approach for alleviating uncontrolled perineal pain and as an attempt to eradicate a tumour that had been dormant for a decade in a patient with favourable overall health.

This case highlights the unusual late presentation of urothelial carcinoma involving the penis, occurring 10 years after a RC. It underscores the importance of close and long‐term follow‐up, regardless of the time elapsed since the initial treatment. Thorough physical examination remains essential as such lesions may go unnoticed and are often associated with poor prognosis. Additionally, distinguishing between urethral recurrence and metastatic disease is crucial as it may have important implications for patient management.

## Funding

This study did not receive any financial grants.

## Disclosure

All scientific content, data interpretation and conclusions are the sole responsibility of the authors. The use of these tools has been reviewed and approved by all co‐authors.

## Conflicts of Interest

The authors declare no conflicts of interest.

## Data Availability

The data supporting the findings of this study are available upon request from the authors.
